# Head and neck paragangliomas: diffusion weighted and dynamic contrast enhanced magnetic resonance imaging characteristics

**DOI:** 10.1186/s12880-016-0114-3

**Published:** 2016-02-01

**Authors:** Ying Yuan, Huimin Shi, Xiaofeng Tao

**Affiliations:** Department of Radiology, Shanghai Ninth People’s Hospital, Affiliated to JiaoTong University School of Medicine, 639 Zhizaoju Road, Shanghai, 200011 China

## Abstract

**Background:**

To determine the feature values of head and neck paragangliomas on diffusion-weighted imaging (DWI) and dynamic contrast enhanced (DCE) magnetic resonance imaging (MRI).

**Methods:**

Patients with primary head and neck paraganglioma who underwent both DWI and DCE-MRI before treatment between January 2010 and June 2013 were identified. Two radiologists assessed apparent diffusion coefficient (ADC) values on DWI and kinetic characteristics on DCE-MRI. The time intensity curves (TICs) and dynamic parameters, including peak height (PH), maximum enhancement ratio (ER_max_), time to peak enhancement (T_peak_) and maximum rise slope (Slope_max_), were generated and evaluated.

**Results:**

Ten patients with head and neck paraganglioma were retrospectively analyzed. On conventional MRI, the tumors demonstrated as well-circumscribed, strongly enhanced lesions. Mean ADC value of the lesions was 1.12 ± 0.15 × 10^−3^ mm^2^/s. The TICs demonstrated washout pattern (type-III) in all lesions. The mean PH, ER_max_, T_peak_ and Slope_max_ value was 121.24 ± 63.99, 193.79 ± 67.18, 8.16 ± 3.29 and 25.42 ± 7.91, respectively.

**Conclusions:**

Head and neck paragangliomas demonstrate distinctive DWI and DCE-MRI results than for other benign tumors which should be taken into account in further evaluation of MRI on head and neck lesions.

## Background

Head and neck paragangliomas are rare tumors originating from the parasympathetic nervous system. They mostly occur at the carotid bifurcation (carotid body tumors), jugular bulb (jugular paraganglioma), vagus nerve (vagal paraganglioma), middle ear mucosa (tympanic paraganglioma) and larynx (laryngeal paraganglia). Less common sites include the sella turcica, pineal gland, cavernous sinus, larynx, orbit, thyroid gland, nasopharynx, mandible, soft palate, face, and cheek [1]. Computed tomography (CT) and magnetic resonance imaging (MRI) have evolved as effective non-invasive imaging modalities for detection and evaluation of paragangliomas. MRI has advantages over CT in better soft tissue contrast, higher intrinsic flow sensitivity and non-ionizing. As reported, characteristic imaging findings were described as well-circumscribed, strongly enhanced masses with “salt-and-pepper” appearance [2–5].

MRI methods, such as diffusion-weighted imaging (DWI) and dynamic contrast enhanced (DCE) MRI, as well as other functional imaging modalities have been showing great potential in oncologic applications of head and neck tumor [6–9]. DWI depicts the brownian motion of water molecules within the tissue, quantified with apparent diffusion coefficient (ADC). DCE-MRI allows in vivo imaging of the physiology of microcirculation and provides information related to the vascularity [10]. However, previous MRI studies of head and neck paragangliomas were mainly focused on conventional MRI and rarely assessed the application of DWI and DCE-MRI. Therefore, the aim of the current study was to determine the MRI characteristics of head and neck paragangliomas on: (1) the range of ADC value; and (2) the pattern of time intensity curves (TICs) and dynamic parameters generated from TICs, including peak height (PH), maximum enhancement ratio (ER_max_), time to peak enhancement (T_peak_) and maximum rise slope (Slope_max_).

## Methods

### Patients

A retrospective review of MRI findings was performed in patients with histopathologically proved head and neck paragangliomas between January 1, 2010 and June 30, 2013. All included patients underwent both conventional MRI, DWI and DCE-MRI within 7 days before surgery and biopsy for histopathological confirmation. Patients were excluded in any of the following conditions: (1) with artifacts interfering the diagnosis; (2) a head and neck cancer was previously diagnosed; or (3) with previous surgical procedures or radiation in head and neck region. This retrospective study was approved by institutional review board of Shanghai Ninth People’s Hospital.

### MRI acquisition

All MRI examinations were performed using a 1.5-T scanner (Signa Excite; GE Medical Systems, Milwaukee, WI, USA) with a head and neck array coil. All patients were placed in the magnet in the supine position. The conventional MRI consisted of axial T1-weighted (T1W) sequence, axial and coronal T2-weighted (T2W) sequences. DWI was performed using a single-shot spin echo echo planar imaging (SE-EPI) sequence. Sensitizing diffusion gradients were applied sequentially in the x, y, and z direction with *b* values set at 0 and 1000 s/mm^2^. The DCE-MRI was obtained with fast spoiled gradient echo (FSPGR) acquisition technique. The sections were acquired in the axial plane, centered on the mass. Gadopentetate dimeglumine (Magnevist, Schering, Berlin, Germany) was intravenously bolus injected via a power injector with a flow rate of 2.0 mL/s at the dose of 0.1 mmol/kg of body weight, followed by a 20 mL saline flush. DCE-MRI was sequentially obtained for 40 dynamic phases for each investigation. Conventional post-contrast T1W sequences were also performed, with fat suppression in coronal sequences. DWI and DCE-MRI images were post-processed and statistically analyzed in the current study. Details of the MRI protocol are provided in Table 1.

**Table 1:** MR imaging protocol

Sequences	T1WI	T2WI	T2WI	DWI	DCE	T1WI^a^	T1WI^a^
Plane	Axial	Axial	Coronal	Axial	Axial	Axial	Coronal
TR (ms)	600	4200	3700	2775	4.8	540	540
TE (ms)	9.9	94.6	81.4	70	2.2	8.8	9.4
NEX	2	2	3	8	1	2	2
Slice thickness/gap (mm)	5/1	5/1	5/1	5/0.5	5/0.5	5/1	5/1
Matrix (mm)	288 × 192	320 × 224	320 × 224	128 × 128	128 × 128	288 × 192	288 × 192
FOV (cm)	24 × 24	24 × 24	24 × 24	24 × 24	24 × 24	24 × 24	22 × 22
FS	No	No	Yes		Yes	No	Yes

*DCE* dynamic contrast-enhanced, *DWI* diffusion weighted imaging, *FOV* field of view, *NEX* number of excitations, *T1WI* T1-weighted imaging, *T2WI* T2-weighted imaging, *TE* echo time (msec), *TR* repetition time

^a^ contrast enhanced sequences

### MRI interpretation

All MR images were processed by two radiologists (Y.Y., H.-M.S.; with 8 and 25 years of experience in head and neck MR imaging, respectively). Blinded to clinical information, imaging results from other modalities and histopathologic results, two radiologists selected representative regions of interest (ROIs) on DWI and DCE-MRI by consensus. Using the post processing software Functool from GE, ADC maps were automatically generated. ADC values were extracted from ADC maps. DWI was evaluated by drawing freehand ROI in lesions over the axial slice with the largest tumor area, taking care to exclude obvious necrotic or non-perfused areas by visual correlation with pre- and post-contrasted T1W images. ADC values were manually measured three times in each lesion, and average ADC value was obtained. ROIs of DCE-MRI were acquired with the following method. After a color-coded mapping of the mass, we placed 2 mm × 2 mm ROIs over the entire lesion on multiple slices. The TIC of each ROI was generated. By comparing the TIC from each ROI, the ROI that demonstrated the greatest degree of enhancement was selected as representation for the lesion. The detail method was described in our previous studies [11, 12]. The TICs (axis coordinate, time; vertical, signal intensity) were categorized as three patterns: (1) the persistent pattern (type-I) with straight or curved line and continuous enhancement over the entire dynamic study; (2) the plateau pattern (type-II) with a relatively prominent increase slope and a final intensity 90–100 % of peak grade; (3) the washout pattern (type-III) with a rapid increase slope and a final intensity lower than 90 % of peak grade. From each TIC, the signal intensity (SI_pre_, SI_max_) and time (T_pre_, T_peak_) were derived. SI_pre_ was defined as the pre-contrast signal intensity. SI_max_ was the signal intensity at maximal contrast enhancement. T_pre_ and T_peak_ were the time corresponding to the SI_pre_ and SI_max_. Other parameters, such as PH, ER_max_ and Slope_max_, were calculated upon the following formulas [11, 12]:

$$ PH=S{I}_{max}\hbox{---} S{I}_{pre} $$

$$ E{R}_{\max }=\frac{S{I}_{max}-S{I}_{pre}}{S{I}_{pre}}\times 100 $$

$$ Slop{e}_{\max }=\frac{S{I}_{max}-S{I}_{pre}}{S{I}_{pre}\times \left({T}_{peak}-{T}_{pre}\right)}\times 100 $$

Statistical analysis was carried out using STATA version 12.0 (CollegeStation, TX). *P* < 0.05 was considered as statistically significant.

## Results

**Fig. 1:**
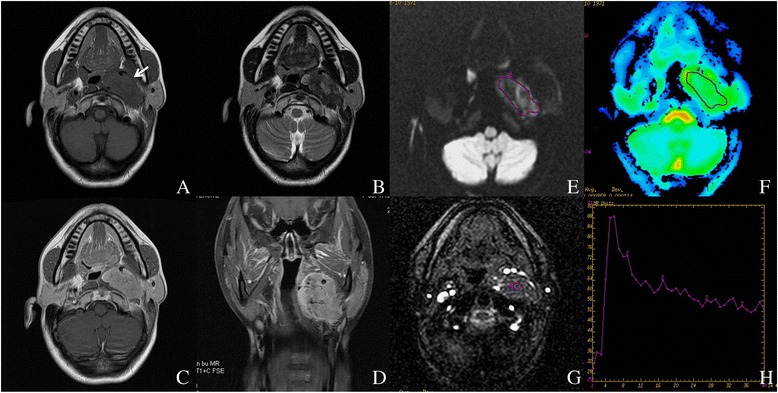
Left carotid body tumor in a 38-year-old female. **a**, **b** Non-enhanced axial T1W and T2W images. **c** Contrast enhanced axial T1W image. **d** Contrast enhanced coronal T1W image with fat suppression. **e**, **f** ROI on DWI (*b* value = 1000 s/mm^2^) and corresponding ADC map. The average ADC value of this lesion was 0.928 × 10^−3^ mm^2^/s. **g**, **h** DCE-MRI and corresponding TIC, which showed a washout pattern

**Fig. 2:**
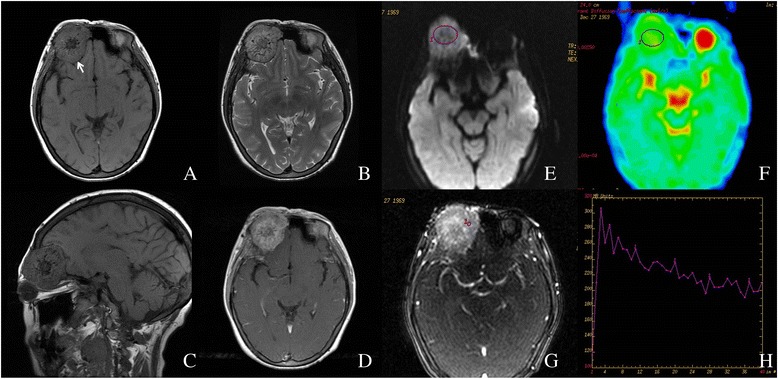
Right orbital paraganglioma in a 36-year-old female. **a**, **b** Non-enhanced axial T1W and T2W MR images. **c** Non-enhanced saggital T2W MR image. **d** Contrast enhanced axial T1W image. **e**, **f** ROI on DWI (*b* value = 1000 s/mm^2^) and corresponding ADC map. The average ADC value of this lesion was 1.196 × 10^−3^ mm^2^/s. **g**, **h** DCE-MRI and corresponding TIC, which showed a washout pattern

Ten patients with untreated head and neck paragangliomas (three male, seven female; age 36.6 ± 15.02 years) were included. DWI and DCE-MRI were performed in all patients. Carotid body tumors were diagnosed in eight patients (8/10, 80 %). Other locations included the jugular fossa (*n* = 1) and orbit (*n* = 1). The tumor size at diagnosis was 4.42 ± 1.65 cm. The radiological findings of each patient are listed in Table 2. On conventional MRI, the tumors demonstrated as well circumscribed and intensely enhanced masses with marked internal vascularity that appeared as multiple signal voids. The mean ADC value was 1.12 ± 0.15 × 10^−3^ mm^2^/s, significantly higher contrasted to adjacent muscle (0.74 ± 0.06 × 10^−3^ mm^2^/s, *P* = 0.0006). The TIC curve unexceptionally demonstrated washout pattern (type-III) in the ten lesions. The mean PH, ER_max_, T_peak_ and Slope_max_ value was 121.24 ± 63.99, 193.79 ± 67.18, 8.16 ± 3.29 and 25.42 ± 7.91, respectively. MRI images of representative cases are shown in Figs. 1 and 2.

**Table 2:** Radiological findings of patients with head and neck paragangliomas

No.	Gender	Age (years)	Location	Size^a^ (mm)	ADC (×10^−3^ mm^2^/s)	TIC pattern	PH	ER_max_	T_peak_	Slope_max_
1	female	38	left carotid	5.0	0.928	washout	55.9	191.4	25.8	7.4
2	female	38	right carotid	1.2	0.834	washout	54.1	185.3	23.3	8.0
3	female	53	right carotid	6.0	1.026	washout	126.4	220.2	20.6	10.7
4	female	36	right orbital	4.8	1.196	washout	189.5	162.0	11.6	14.0
5	female	55	left carotid	4.3	1.257	washout	89.8	95.0	19.3	4.9
6	female	16	right carotid	4.4	1.227	washout	133.8	302.0	29	10.4
7	female	44	left jugular bulb	3.7	1.045	washout	254	107.5	25.8	4.2
8	male	16	left carotid	3.5	1.325	washout	118.3	238.0	31.6	7.5
9	male	19	left carotid	7.5	1.14	washout	135.7	276.4	25.8	10.7
10	male	51	right carotid	3.8	1.143	washout	54.9	160.1	41.4	3.9

*ADC* apparent diffusion coefficient, *TIC* the time intensity curve

^a^ The longest diameter on the axial slices

## Discussion

Head and neck paragangliomas are hypervascular benign neoplasms derived from the embryologic neural crest. They account for 0.6 % of all neoplasms in the head and neck region. The most common locations are carotid space and jugular fossa. In the current study, eight of the ten paragangliomas were from the carotid body. The other two lesions were respectively in the jugular fossa and the left orbit, which is a rare location to our knowledge [1, 13]. As reported, 10 % tumors may be multicentric in origin and approximately 6 to 19 % are malignant, as evidenced by the presence of regional and/or distant metastases [14, 15]. In the current study, there was no multifocal or metastatic lesion, probably due to the small amount of patients. The patients were all sporadic cases without a familial predisposition.

Contrast-enhanced CT and MRI have evolved as effective imaging modalities for detection of paragangliomas. The lesions have conventional MRI findings of well-circumscribed and strongly enhancing masses indicating abundant blood supply, which was also proved in our study. Salt-and-pepper appearance of the tumor, expansive growth and remodeling of adjacent bony structures are also diagnostic [16]. DWI has shown promise in the diagnosis, staging and prognostic evaluation of head and neck tumors [17]. Previous studies have confirmed a significant difference in ADC values between benign and malignant lesions [18, 19]. Lower ADC values have been reported in the head and neck region for most malignant lesions, compared with those of benign lesions. Optimal ADC threshold values of 1.3 × 10^−3^ mm^2^/s (*b* = 800 s/mm^2^, 3-T MR unit) [18] and 1.25 × 10^−3^ mm^2^/s (*b* = 500, 1000 s/mm^2^, 1.5-T MR unit) [19] were suggested to help distinguish benign from malignant lesions. The mean ADC value was of the head and neck paragangliomas under study 1.12 ± 0.15 × 10^−3^ mm^2^/s. Eight tumors showed ADC value lower than 1.25 × 10^−3^ mm^2^/s, which could have suggested a malignant nature according to abovementioned criteria [18, 19]. Other DWI results here! The relatively lower ADC value detected in the current study than other benign tumor as previously reported might be attributed to the inner texture of the lesion. Solid tumors without apoptosis or necrosis show higher signal intensity and lower ADC value [17]. Therefore, although DWI shows the potential in characterizing and differentiating head and neck lesions, given the heterogeneous of benign and malignant lesions, there will clearly be exceptions and overlaps. When dealing with lesions with lower ADC value in head and neck region, radiologists should carefully exclude the possibility of paraganglioma before giving it a malignant diagnosis, especially for those lesions without typical conventional MRI characteristics.

DCE-MRI has been employed for tumor detection and characterization as well [20]. Malignant tumors tend to exhibit strong initial signal increase followed by washout effect, which is equivalent to washout pattern (type-III) in our study, whereas benign lesions mostly demonstrate slow initial signal enhancement combined with continuous signal increase (the persistent pattern). A plateau curve can be seen in both benign and malignant lesions. However, some malignant processes can mimic benign contrast kinetics and vice versa [21]. It is to be noted in the current study that the TICs unexceptionally demonstrated washout pattern in all cases. In a previously conducted study for visualization of paragangliomas with magnetic resonance projection angiography (MRPA), an early and rapid enhancement after contrast administration and a washout effect after maximum of intensity was also detected [22]. The “washout” of intensity is usually attributed to malignant arteriovenous anastomoses, neovascular capillary hyperpermeability and the high flow rate in the newly formed capillaries [23, 24]. Correspondingly, in the current study, the TIC pattern might be related to the typical angiographic appearance of a paraganglioma as a hypervascular mass with enlarged feeding arteries, intense tumor blush and early draining veins [16, 25], which is distinctive and not commonly seen in benign tumors.” DWI and DCE-MRI could indicate the functional characteristics of tumor with regard to the texture and blood supply; however, these methods cannot be used exclusively with the same standard and threshold for differentiate benign from malignant tumors, as also reported in a systemic review of salivary gland tumors [26]. The washout TIC together with conventional MRI characteristics of well-defined margin and strong enhancement as well as a lower ADC value (mostly lower than 1.25 × 10^−3^ mm^2^/s in the current study) would direct to a diagnosis of paraganglioma in head and neck region, which is especially distinct in ADC and TIC results than other benign tumors.

Our study has some limitations. First, we included a relatively small number of cases, not only due to the low incidence rate of the disease, but also the inclusion criteria of the study. Major treatments for head and neck paragangliomas include observation and clinical follow-up, surgical excision, embolism and radiotherapy. We only included those histopathologically confirmed by specimens obtained at biopsy or surgery. Moreover, the inlcuded patients should undergo preoperative DWI and DCE-MRI. In spite of the small amount of cases, the results of the current study could still deliver a message that head and neck paragangliomas demonstrate distinctive DWI and DCE-MRI results compared to other benign lesions. Second, the selection of ROIs would affect the reproducibility of ADC values and kinetic curves. We chose to acquire a mean ADC by drawing three times of freehand ROIs over the axial slice with the largest tumor area, taking care to exclude obvious necrotic or non-perfused areas, in order to acquire ADC values most representative of the solid components in tumors. More sophisticated techniques for quantitative evaluation of DWI have been introduced, including histogram analysis and voxel-by-voxel changes; however, these techniques are mostly not yet available to clinical radiologists [17]. As for DCE-MRI, as described in our previous studies [11, 12], representative ROIs were defined as those demonstrated the greatest degree of peak enhancement. Two readers interpreted the images in consensus and inter-observer reliability could not be calculated, which might be another limitation of the study. Intravoxel incoherent motion (IVIM) MRI is a technique with the potential for simultaneously assessing both tissue perfusion and diffusion by using a single DWI. Recently, several attempts have been made to determine the feasibility of IVIM for differential diagnosing head and neck tumors [27, 28] and evaluating head and neck squamous cell carcinoma (HNSCC) [29], by comparing and combining with DWI and DCE-MRI results.

## Conclusions

Head and neck paragangliomas demonstrate distinctive features on DWI and DCE-MRI than other benign tumors. In addition to the morphological features, a relatively lower ADC value and washout TIC would support the diagnosis, which is otherwise more supportive for a malignant nature. Therefore, the interpretation of MRI in head and neck lesions should take into account the possible exceptions. Further studies are needed to be performed in larger group of patients and to compare the differential ability of DWI and DCE-MRI between paragangliomas and other head and neck lesions.

## Abbreviations

ADC: apparent diffusion coefficient
CT: computed tomography
DCE: dynamic contrast enhanced
DW: diffusion-weighted
ER_max_: maximum enhancement ratio
FSPGR: fast spoiled gradient echo
IVIM: intravoxel incoherent motion
MR: magnetic resonance
PH: peak height
ROI: regions of interest
SE-EPI: single-shot spin echo echo planar imaging
Slope_max_: maximum rise slope
TIC: time intensity curve
T_peak_: time to peak enhancement
T1W: T1-weighted
T2W: T2-weighted
